# A Regulatory Polymorphism in *HAVCR2* Modulates Susceptibility to HIV-1 Infection

**DOI:** 10.1371/journal.pone.0106442

**Published:** 2014-09-02

**Authors:** Manuela Sironi, Mara Biasin, Federica Gnudi, Rachele Cagliani, Irma Saulle, Diego Forni, Veronica Rainone, Daria Trabattoni, Micaela Garziano, Francesco Mazzotta, Luis Miguel Real, Antonio Rivero-Juarez, Antonio Caruz, Sergio Lo Caputo, Mario Clerici

**Affiliations:** 1 Bioinformatics, Scientific Institute IRCCS E. MEDEA, Bosisio Parini, Italy; 2 Department of Biomedical and Clinical Sciences, University of Milan, Milan, Italy; 3 Infectious Disease Unit, S. Maria Annunziata Hospital Florence, Florence, Italy; 4 Infectious Diseases and Microbiology Clinical Unit, Valme Hospital, Seville, Spain; 5 Maimonides Institut for Biomedical Research (IMIBIC)-Reina Sofia Universitary Hospital-University of Cordoba, Cordoba, Spain; 6 Immunogenetics Unit, Department of Experimental Biology, University of Jaen, Jaen, Spain; 7 Chair of Immunology, Department of Physiopathology and Transplantation, University of Milan, Milan, Italy; 8 Don C. Gnocchi Foundation ONLUS, IRCCS, Milan, Italy; University of Alabama at Birmingham, United States of America

## Abstract

The *HAVCR2* gene encodes TIM-3, an immunoglobulin superfamily member expressed by exhausted CD8+ T cells during chronic viral infection. We investigated whether genetic variation at HAVCR2 modulates the susceptibility to HIV-1 acquisition; specifically we focused on a 3′ UTR variant (rs4704846, A/G) that represents a natural selection target. We genotyped rs4704846 in three independent cohorts of HIV-1 exposed seronegative (HESN) individuals with different geographic origin (Italy and Spain) and distinct route of exposure to HIV-1 (sexual and injection drug use). Matched HIV-1 positive subjects and healthy controls were also analyzed. In all case-control cohorts the minor G allele at rs4704846 was more common in HIV-1 infected individuals than in HESN, with healthy controls showing intermediate frequency. Results from the three association analyses were combined through a random effect meta-analysis, which revealed no heterogeneity among samples (Cochrane's Q, p value =  0.89, I^2^ =  0) and yielded a p value of 6.8 ×10^−4^. The minor G allele at rs4704846 was found to increase *HAVCR2* expression after in vitro HIV-1 infection. Thus, a positively selected polymorphism in the 3′ UTR, which modulates HAVCR2 expression, is associated with the susceptibility to HIV-1 infection. These data warrant further investigation into the role of TIM-3 in the prevention and treatment of HIV-1/AIDS.

## Introduction

TIM-3 (T cell immunoglobulin and mucin domain-containing molecule 3) is an immunoglobulin superfamily member encoded in humans by the *HAVCR2* (hepatitis A virus cellular receptor 2) gene. Initially identified as a marker of IFN-γ-producing CD4+ Th1 and CD8+ Tc1 cells [1], TIM-3 was more recently shown to be expressed by several other immune cell types including NK/NTK, macrophages/monocytes, and dendritic cells [2]. The protein product of *HAVCR2* can bind both galectin 9 (Gal-9) and phosphatidylserine [2]. Engagement of TIM-3 by Gal-9 on T cells induces cell death and promotes peripheral tolerance [2]. Thus, TIM-3 plays an important role in the negative regulation of T-cell mediated responses, and abrogation of its signaling increases the secretion of IFN-γ by activated human T cells [3]. Recent evidences have indicated that expression of TIM-3 marks a population of exhausted CD8+ T cells during chronic viral infection [2]. Specifically, in progressive HIV-1 infection TIM-3 defines an abundant population of CD8+ T cells and its expression correlates positively with viral load and inversely with CD4+ T cell counts [4]. The loss of proliferative activity of HIV-specific TIM-3-expressing CD8+ cells is partially mediated by the interaction with Gal-9 on T_reg_ cells and is modulated by *HLA-B* allelic status [5]. Despite these observations, the role of TIM-3 in HIV-1 acquisition has never been analyzed.

We have reported that a variant located in the 3′UTR of *HAVCR2* (rs4704846) has been a target of natural selection in human populations and suggested that the selective pressure is accounted for by infectious agents [6]. In line with this view, a SNP (rs3087616) located 62 bp apart and in full linkage disequilibrium with rs4704846 (r^2^ =  1 in Europeans) has recently been shown to act as an expression QTL (eQTL) in CD14+ monocytes [7]. Given the central role of TIM-3 in viral infection [2], and because evolutionary and eQTL analyses point to rs4704846 (or a closely linked variant) as a functional polymorphism, we investigated whether this SNP modulates the susceptibility to HIV-1 infection.

## Materials and Methods

### Ethics statement

The study was designed and performed according to the Helsinki declaration and was approved by the Ethics Committees of following Institutions: University of Jaen, Valme Hospital (Seville), Reina Sofia Universitary Hospital (Cordoba), and S. Maria Annunziata Hospital (Florence). All patients and healthy blood donors provided written informed consent to participate in this study.

### Subject cohorts

Ninety-three Italian HESN that had been exposed to the virus through unprotected sexual intercourse (SexExp-HESN) and 87 HIV-1-infected subjects were recruited at the S. M. Annunziata Hospital in Florence, Italy; all of them were Italian of European origin. Inclusion criteria for HESN were a history of multiple unprotected sexual episodes for more than 4 years at the time of the enrolment, with at least 3 episodes of at-risk intercourse within 4 months prior to study entry, and an average of 30 (range, 18 to >100) reported unprotected sexual contacts per year [8]. All individuals (SexExp-HESN and HIV-1 infected) had been longitudinally followed for >4 years before the study by the Department of Obstetrics and Gynecology of the S. M. Annunziata Hospital. This allowed us to exclude from the study HESN and HIV-1 infected subjects in whom sexually transmitted diseases or any other pathology were reported during that time period. The range of CD4 counts in HIV-1 infected patients were 36–850 cells/ml, and viral loads were >50–750000 copies/ml. All of the patients were receiving highly active antiretroviral therapy (HAART) at the time of the study.

Thirty-eight Spanish HESN that had been exposed to the virus through unprotected sexual intercourse (SexExp-HESN) were recruited as well. These subjects are female partners of HIV-1 infected patients that were treatment-naive and viremic. In this case, mean number of unprotected sexual intercourse per year was 110 and the mean number of years of unprotected sex was 5 (range 3–17 years). Healthy controls (HC, n = 77) were anonymous blood donors from the City of Jaen Hospital in Jaen, Spain.

Finally, we recruited 190 males exposed to HIV-1 infection by injection drug use (IDU) and enrolled in prospective cohort studies in Spain (Valme Hospital, Sevilla) who had shared needles for >3 months. Concurrent markers of hepatitis C virus (HCV) infection, the most chronic viral infection transmitted by sharing needles, were present in 100% of IDU subjects. These values are significantly higher than the reported HCV prevalence of 1%–2% for the general population in Spain. Ninety-three of these subjects were HIV-1 negative (IDU-HESN), 97 were HIV-1 positive (IDU-CTR). The mean of CD4 cells in HIV-1 infected patients was 648±408 mm^3^, and viral loads were undetectable in 81% of the patients. 87% of these patients were receiving HAART at the time of the study.

All subjects were Spanish of European origin. The main epidemiological characteristics of the populations studied are detailed in Table 1.

**Table 1:** Characteristic of the study cohorts.

	HESN	HIV-1 infected	HESN	HC (HIV-1 negative)	HESN	HIV-1 infected
**Cohort Origin**	Italy		Spain		Spain	
**Exposure**	Heterosexual		Heterosexual		IDU	
**Total Number**	93	87	38	77	93	97
**Age (mean± SD** a **)**	49±8.6	47±5.9	40±6.9	39±8.9	32±7.6	34±8.6
**Gender (male/female)**	30/63	31/56	0/38	0/77	93/0	97/0
**Time of injection drug use (months)**	-		-		52±23.2	
**Mean number of unprotected sexual intercourse per year**	30		110		-	

a SD: standard deviation.

### Genotyping and statistical analysis

Genomic DNA was used as template for PCR amplification using TaqMan probes specifically designed to perform a SNP genotyping assay for rs4704846 (G/A) and using the allelic discrimination real-time PCR method. Assays were performed in 10 µl reactions, using TaqMan Genotyping Master Mix on 96-well plates using a ABI 7000 instrument (Applied Biosystems Foster City, CA, USA). The variant complied to Hardy-Weinberg equilibrium in all samples. Genetic association analyses were performed by logistic regression using an additive model, and results from the three cohorts were combined using a random-effect meta-analysis; all analyses were performed using PLINK [9]. Linkage disequilibrium analyses were performed using Haploview (v. 4.1) [10] and blocks were identified through the confidence interval algorithm implemented in the software [11].

### PBMC isolation, HIV-1 infection, and transcript quantification

Whole blood was collected from 40 healthy volunteers by venipuncture in Vacutainer tubes containing EDTA (Becton Dickinson), and PBMCs were separated on lymphocyte separation medium (Organon Teknica, Malvern, PA). PBMCs (10×10^6^ cells/ml) were cultured for 2 days at 37°C and 5% CO2 in RPMI 1640 containing FBS (20%), PHA (7.5 ìg/ml), and IL-2 (15 ng/ml). After viability assessment, 2.5×10^6^ cells were resuspended in medium containing or not containing 1 ng HIV-1Ba-L/1×10^6^ PBMC and incubated for 3 h at 37°C. Cells were then washed and resuspended in 3 ml complete medium with IL-2 (15 ng/ml). Cells were plated in 24-well tissue culture plates and incubated at 37°C and 5% CO2. After 3 days, 2.5×10^5^ PBMCs were collected for gene expression analysis. RNA was extracted from cultured PBMCs and from HIV-1-infected PBMCs by using the acid guanidium thiocyanate–phenol–chloroform method. The RNA was treated with RNase-free DNase (New England Biolabs, Ipswich, MA). One microgram of RNA was reverse transcribed into first-strand cDNA in a 20-ìl final volume containing 1 ìM random hexanucleotide primers, 1 ìM oligonucleotide, and 200 U Moloney murine leukemia virus reverse transcriptase (Promega, Madison, WI). cDNA quantification for *HAVCR2* and *GAPDH* was performed by real-time PCR (DNA Engine Opticon 2; MJ Research, Ramsey, NJ). Reactions were performed using a SYBR Green PCR mix (5 prime, Gaithersburg, MD). Results were expressed as ÄÄCt and presented as ratios between the target gene and the GAPDH housekeeping mRNA.

## Results

**Figure 1:**
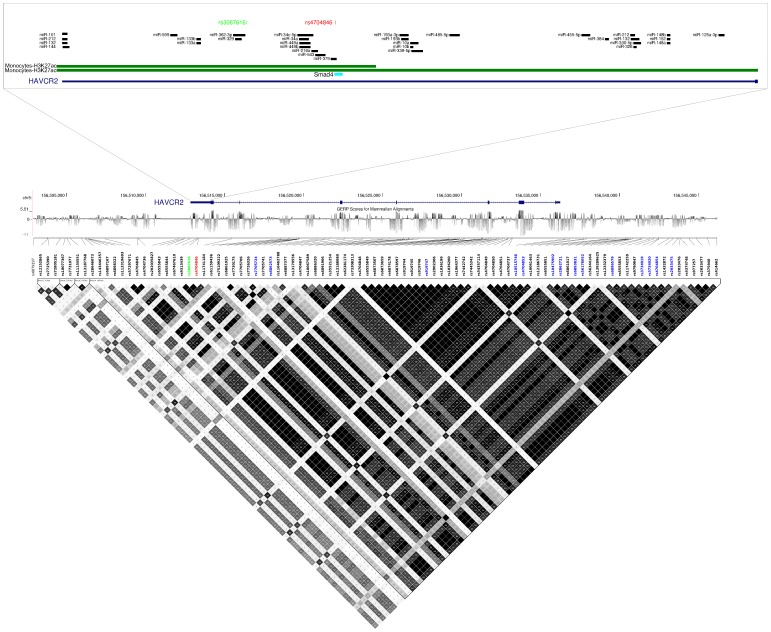
LD analysis and functional annotation. The *HAVCR2* gene region is shown within the UCSC Genome Browser view. Variants that have been described as eQTLs in monocytes and lymphoblastoid cell lines are in green and blue, respectively. The variant we analyzed is in red. The location of predicted functional elements and microRNA binding sites is also shown. LD analysis is reported in the bottom panel; data refer to Europeans and derive from the 1000 Genomes Projects Phase I data for Europeans (CEU) [12]. r^2^ was calculated with the Haploview software and blocks were identified through the implemented confidence interval algorithm (see methods).

As mentioned above, rs4704846 is located in the 3′UTR of *HAVCR2*. The ancestral minor G allele displays a frequency of 0.39, 0.20, and 0.01 in Africans (Yoruba), Europeans and Japanese plus Chinese, respectively, as assessed by the 1000 Genomes Project data [12]. Analysis of linkage disequilibrium (LD) in Europeans along *HAVCR2* indicated that rs4704846 lies within an LD block that also includes rs3087616, an eQTL in naive CD14+ monocytes. The variant is also in full LD with several SNPs that were described as eQTLs in lymphoblastoid cell lines [13], [14] (Fig. 1). The 3′UTR of *HAVCR2* is moderately conserved in mammals, as assessed through the GERP (Genomic Evolutionary Rate Profiling) score [15] (Fig. 1); prediction of regulatory motifs using rSNPBase [16] indicated that rs4704846 maps to regions showing H3K27ac histone marks (usually associated with active regulatory elements) in CD14+ monocytes and several microRNA binding sites are observed across the entire 3′ UTR (microRNa.org, http://www.microrna.org/microrna/home.do). The presence of regulatory motifs was further analyzed using HaploReg [17]: rs4704846 was found to affect a Smad4 binding site (Fig. 1). Smad4 is a mediator of TGF-beta signaling and its expression after SIV infection differs in rhesus macaque and African green monkeys, which are differentially susceptible to SIV-mediated immunopathology [18].

To explore the role of rs4704846 in HIV-1 acquisition, we genotyped this variant in a well characterized cohort of 93 heterosexual Italian subjects who have a history of unprotected sex with their seropositive partners (sex-exposed HESN, SexExp-HESN). The allele frequency of rs4704846 in these subjects was compared to that observed in a sample of 87 Italian HIV-1 positive individuals. A significant difference was observed, with the minor G allele being much more common in HIV-1 infected (0.25) compared to SexExp-HESN (0.14) (Table 2). The frequency of the G allele in Italians is 0.21, as determined by the 1000 Genomes Phase I Project. We genotyped 300 Italian healthy subjects and obtained a similar frequency of 0.20, which is therefore intermediate between HIV-1 infected and SexExp-HESN. A very similar result was obtained in a second and smaller cohort of SexExp-HESN (n = 38) from Spain: in these subjects the frequency of the G allele was 0.12, much lower than in a sample of 77 Spanish healthy controls (HC, frequency = 0.20); due to the small sample size the association p value did not reach statistical significance (Table 2). Finally, to replicate these results, a third HESN population with a different route of exposure to HIV-1 was analyzed. In particular, we recruited 190 Spanish injection drug users (IDU): all of them were HCV-positive, but, whereas 97 subjects were HIV-1 infected (IDU-HIV-1 infected) as well, the remaining 93 individuals tested HIV-1 negative despite multiple exposures through needle sharing (IDU-HESN). Again, the G allele was more common in IDU-HIV-1 infected compared to IDU-HESN with a borderline significance of 0.064.

**Table 2:** Association of rs4704846 with HIV-1 infection susceptibility.

Sample	Minor allele	Allele frequency		Additive model		Additive model(combined)	
		SexExp-HESN	HIV-1 infected	*p* a	OR (95 IC)^b^	*p* c	OR^c^
Italy	G	0.14	0.25	0.015	2.02 (1.15–3.56)		
		SexExp-HESN	HC				
Spain	G	0.12	0.20	0.119	1.92 (0.85–4.36)	6.80 × 10^−4^	1.85
		IDU-HESN	IDU-HIV-1 infected				
Spain	G	0.18	0.25	0.064	1.67 (0.97–2.88)		

a Logistic regression p value for an additive model.

b Odds ratio with 95% confidence intervals.

c Random-effect meta-analysis *p* value (additive model) and OR.

Results from the three association analyses were combined through a random effect meta-analysis, which revealed no heterogeneity among samples (Cochrane's Q p value =  0.89, I^2^ =  0) and yielded a p value of 6.8 ×10^−4^ (Table 2). Overall, these results strongly suggest that the minor G allele of rs4704846 is associated with increased risk of HIV-1 acquisition.

**Figure 2:**
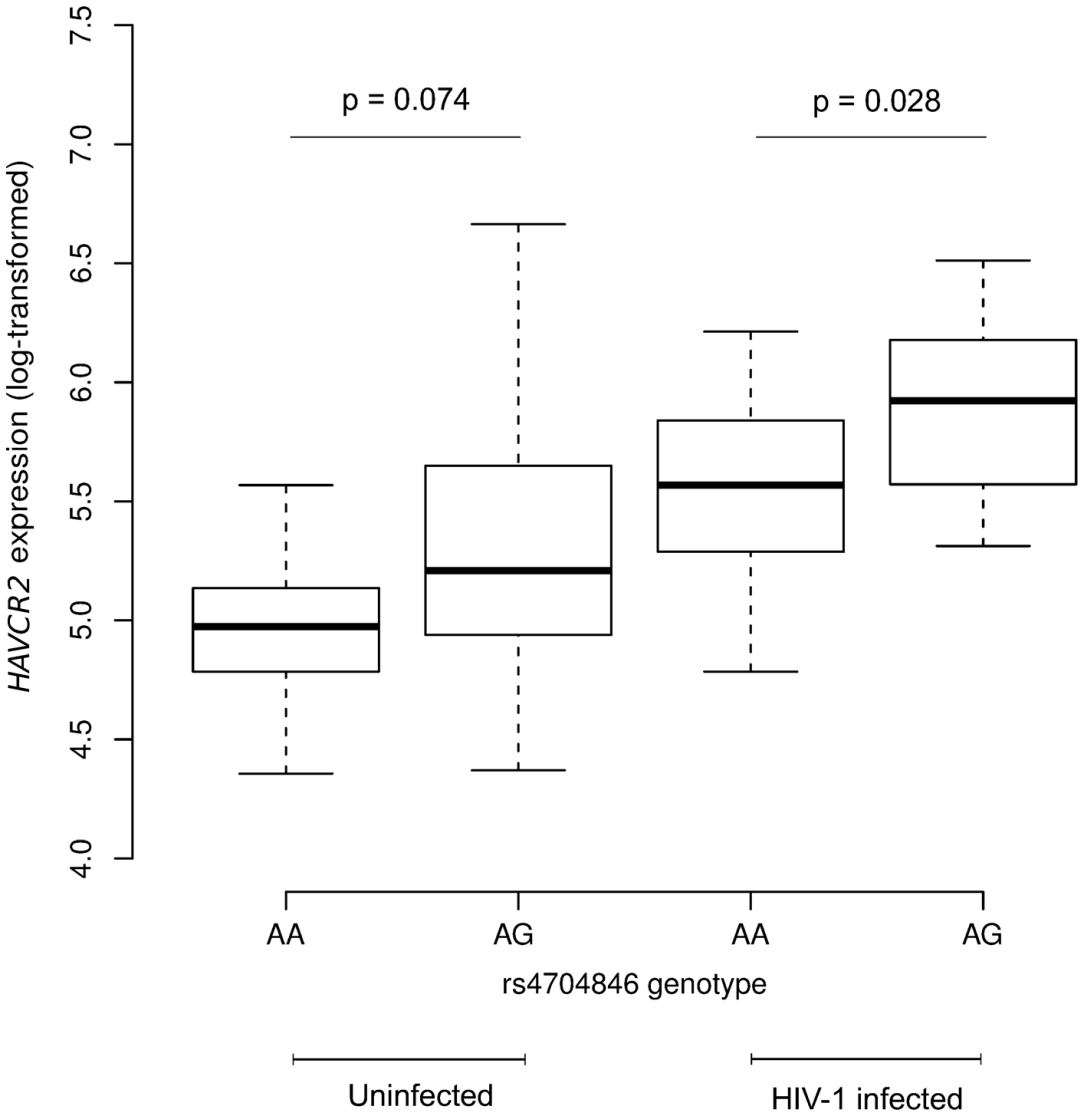
Box-and-whisker plot of *HAVCR2* expression depending on rs4704846 genotype. Data derive from PBMCs from 40 healthy volunteers uninfected or infected with HIV-1. *HAVCR2* transcript levels are log-transformed and shown in standard box-and-whisker plot representation (thick line: median; box: quartiles; whiskers: 1.5 × interquartile range); p values are calculated using the Student's *t*-test.

To assess whether the modulatory effect of rs4704846 on HIV-1 susceptibility is exerted through changes in *HAVCR2* expression, we performed an in vitro infection assay. Specifically, PBMCs from 40 healthy subjects (27 AA, 13 AG) were infected with HIV-1 and *HAVCR2* transcript abundance was quantified by real-time PCR after 3 days. As shown in figure 2, significantly higher *HAVCR2* expression was observed following *in vitro* HIV-1 infection in AG heterozygotes compared to AA homozygotes (Student's *t*-test, p = 0.028) (Fig. 2); the same trend was observed in uninfected PBMCs, although the difference was less marked and did not reach statistical significance (Fig. 2).

## Discussion

We analyzed three independent HESN cohorts and in all cases we observed the same trend: HIV-1 infected subjects are more likely to carry the G allele than HESN, with healthy controls showing intermediate frequency. Therefore, although statistical analysis was fully significant for the Italian cohort only, the meta-analysis we performed revealed no heterogeneity in the effect of rs4704846 among the three samples and yielded a highly significant association result. Indeed, the small sample size of HESN cohorts, which is conceivable given the characteristics of these subjects, make replication across populations and meta-analysis a powerful approach to discover susceptibility variants for HIV-1 infection. We should add that the single case-control samples used either HIV-1 infected or HC subjects as a comparison to HESN; nonetheless, we believe meta-analysis to be appropriate as HIV-1 infection susceptibility is a common condition to most humans [19], [20].

The interaction between TIM-3 and Gal-9 acts to limit the extent of CD8^+^ T cell immunity to HSV infection [21] and over-expression of TIM-3 on CD4+ and CD8+ T cells correlates with diseases progression in chronic hepatitis B infection [22]. Likewise, the chronic persistence of HIV-1 is associated with the increased expression of TIM-3 on CD4+ and virus-specific CD8+ T cells [4]. Results herein fit within this scenario by showing that the expression level of *HAVCR2* following infection is at least partially determined by a polymorphism that also affects susceptibility to HIV-1. Although protein and RNA levels do not correlate perfectly in humans[23], it is conceivable that allelic status at rs4704846 also affects TIM-3 abundance in PBMCs.

Natural selection targets variants with a phenotypic effect and acts in response to specific selective pressures. The major allele of rs4704846, which associates with lower susceptibility to HIV-1 acquisition, has been driven to high frequency in human populations by natural selection [6]. Because its appearance as a human pathogen is recent, HIV-1 cannot be regarded as the underlying selective pressure. Nonetheless, as mentioned above, TIM-3 dysregulation has been associated with chronic and acute infections with other viral species [4], [22], [24], suggesting that extant or extinct pathogens drove the frequency increase of the protective allele. The location of rs4704846 in the 3′ UTR and its full linkage-disequilibrium with eQTLs in monocytes and lymphoblastoid cell lines are in agreement with the effect we observed on *HAVCR2* expression; nonetheless, the underlying molecular mechanism(s) (miRNA-mediated regulation, transcription factor binding site alteration or other) remains to be determined. Although replication in additional independent samples will be necessary, data herein warrant further investigation into the role of *HAVCR2* in the prevention and treatment of HIV-1/AIDS.
